# The cost-effectiveness of a culturally tailored parenting program: estimating the value of multiple outcomes

**DOI:** 10.1186/s12962-021-00278-4

**Published:** 2021-04-23

**Authors:** Camilla Nystrand, Filipa Sampaio, Jeffrey S. Hoch, Fatumo Osman, Inna Feldman

**Affiliations:** 1 Department of Public Health and Caring Sciences, Uppsala University, Uppsala, Sweden; 2 Division of Health Policy and Management, Department of Public Health Sciences, University of California Davis School of Medicine, Sacramento, CA USA; 3 School of Education, Health and Social Studies, Dalarna University, Falun, Sweden; 4 Department of Epidemiology and Global Health, Umeå University, Umeå, Sweden

**Keywords:** Child health, Cost-effectiveness, Parenting program

## Abstract

**Background:**

Parenting programs can be economically attractive interventions for improving the mental health of both parents and their children. Few attempts have been made to analyse the value of children’s and parent’s outcomes simultaneously, to provide a qualified support for decision making.

**Methods:**

A within trial cost-effectiveness evaluation was conducted, comparing *Ladnaan*, a culturally tailored parenting program for Somali-born parents, with a waitlist control. Quality-adjusted life years (QALY) for parents were estimated by mapping the General Health Questionnaire-12 to Euroqol’s EQ-5D-3L to retrieve utilities. Behavioural problems in children were measured using the Child Behaviour Checklist (CBCL). Intervention costs were estimated for the trial. A net benefit regression framework was employed to study the cost-effectiveness of the intervention, dealing with multiple effects in the same analysis to estimate different combinations of willingness-to pay (WTP) thresholds.

**Results:**

For a WTP of roughly €300 for a one point improvement in total problems on the CBCL scale (children), *Ladnaan* is cost-effective. In contrast, the WTP would have to be roughly €580,000 per QALY (parents) for it to be cost-effective. Various combinations of WTP values for the two outcomes (i.e., CBCL and QALY) may be used to describe other scenarios where *Ladnaan* is cost-effective.

**Conclusions:**

Decision-makers interested in multiple effects must take into account combinations of effects in relation to budget, in order to obtain cost-effective results. A culturally adapted parenting program may be cost-effective, depending on the primary outcome, or multiple outcomes of interest.

*Trial registration* clinicaltrials.gov, NCT02114593. Registered 15 April 2014—prospectively registered, https://www.clinicaltrials.gov/ct2/results?recrs=&cond=&term=NCT02114593&cntry=&state=&city=&dist=

## Background

Child mental health may have profound consequences for her family, friends, teachers and others [1], including effects on health-related quality of life of primary caregivers and other family members [2]. Bi-directionally, child internalising and externalising problems may be associated to parental depression [3–5]. Targeting any one of these could thus have substantial, multidirectional spillover effects on others.

Child and parent mental health problems also incur large societal costs [6], especially if concurrent [7]. There is a wide range of research on including spillover effects in effectiveness evaluations, including effects on different outcomes related to health, employment and schooling [8]. These attempts have included both generic health outcomes such as quality-adjusted life years (QALY) and clinical, non-generic outcomes. A small but growing body of research has also attempted to include spillover effects in the estimation of the cost-effectiveness of interventions [9]. However, few studies have attempted to simultaneously assess health outcomes that are generic and clinical. This approach is interesting in relation to a decision-maker’s willingness-to-pay (WTP) for different outcomes. For generic health outcomes, national and international health technology assessment organizations recommend levels at which new treatments would be deemed good value for money, i.e. cost-effective [10, 11]. Additionally, the World Health Organization recommends a WTP level of less than three times the national annual gross domestic product per capita [12]. These recommended values could be used as shadow estimates for the WTP for a generic outcome. There are no “general guidelines” for the WTP for clinical outcomes, which are often more intuitive, clinically relevant and collected alongside clinical-trials. It is thus difficult to assess whether an intervention is cost-effective, if we use clinical outcome measures. An approach developed to overcome this difficulty is the incremental net benefit (INB) framework, as it may be applied in the case of clinical outcomes or while combining two different measures of effectiveness [13].

Parenting interventions may effectively prevent or reduce mental health problems in children, as well as improve parental psychosocial well-being, reduce stress and depression [14–18]. As parenting interventions primarily target parental practices and skills, aiming to indirectly change child behaviour, it may be relevant for decision-makers who consider effects on both children’s and parent’s in their priority setting. Parenting interventions have shown similar effects for parents with different ethnic backgrounds [19]. However, participation rate of parents from diverse ethnicities in commonly delivered programs remains low, especially since perception of problems and therefore acknowledgement of needs may differ, creating barriers to service acquisition and delivery [20]. A culturally adapted version of the parenting program Connect [21] has shown promising results for Somali-born parents [22] and their children [23] in Sweden, with high retention rates. However, the economic credentials of a parenting intervention that has been culturally adapted for a specific ethnic group remain unknown.

## Methods

### Aim

The aim of the paper was to determine the cost-effectiveness of a culturally adapted parenting intervention delivered to Somali-born parents, by including two measures of effects, one for children and one for parents, in the same analysis.

### Study design and participants

One hundred and twenty families were randomized to receive *Ladnaan* (n = 60) or assigned to a waitlist control (n = 60). Outcomes of interest were collected by questionnaires at baseline together with an informed consent (all 120 parents), and at two months post intervention completion. In addition, the same data were collected roughly 3 years post intervention; however, most participants in the waitlist control had by then received *Ladnaan*. Both parents were invited to participate if they were (1) Somali-born with children aged 11–16 years and (2) experienced self-reported stress related to parenting. Parents were excluded if they had participated in another parenting program or if they suffered severe mental illness. If parents had more than one child, they had to choose an index child for whom they filled out the questionnaire. Demographic information related to the parents and their children can be found in Table 1. Ethical approval was obtained from the Swedish Regional Ethical Review Board in Uppsala (Dnr 2014/048).

**Table 1:** Sociodemographic information of parents and children in the intervention group and waitlist control group

Variable	Intervention group	Waitlist control
	n (%)	n (%)
Mothers	43 (72)	37 (62)
Fathers	17 (28)	23 (38)
Child of male sex	36 (60)	33 (55)
Mean age parent (SD)	44 (8)	45 (9)
Mean age child (SD)	14 (2)	13 (2)
Years in Sweden (parent)		
1–5	39 (65)	34 (57)
6–9	10 (17)	19 (32)
≥ 10 y	11 (18)	7 (12)

### Intervention

Based on a qualitative study [24], the parenting program Connect was culturally adapted to the needs of the Somali population in Sweden, and renamed *Landaan*. The main adaptation constituted an additional two sessions to the original program, including themes of child rights, parenting styles and information regarding how the social services in Sweden work. These sessions were held as workshops, lectures and discussions through the local authority. *Ladnaan* continued with the 10-session manual-based parenting program Connect [21]. This attachment-based program focuses on the parent–child relationship and dynamics, promoting parents to reflect on how their own emotional responses affect child behaviour. Translation and cultural adaptation of Connect was conducted. Two group leaders held weekly group sessions for 1–2 h, allowing 12–17 parents to participate in each group. The effectiveness of the intervention has previously been evaluated for children [22] and parents [23].

### Health outcomes

#### Outcome #1

The program intended to improve child emotional and behavioural problems by affecting parents’, for instance, sense of competence in parenting. Child problems were measured using the Child Behavior Checklist (CBCL) developed for ages 6 to 18 [25], rated by the parents. Part of the 133 item instrument concerns emotional and behavioural problems, which were used in this study. This composite measure included internalizing (anxious, withdrawn and somatic) problems, externalizing (rule breaking and aggression) problems as well as social and thought problems and difficulties with attention.

#### Outcome #2

Parent mental health was measured using the General Health Questionnaire (GHQ-12) [26], consisting of 12 items, each asking the parent to rate the degree of symptoms from ‘less than usual’ to ‘much more than usual’. An index score was created, summing answers from all items. The individual total score from the GHQ-12 was used to estimate health-related quality of life (HRQoL). A published algorithm was used to map GHQ-12 scores to the Euroqol’s five dimension three level scale (EQ-5D-3L), using Swedish tariffs for the preference weights [27, 28]. The EQ-5D-3L is a widely used preference-based multi-attribute utility instrument, measuring changes in HRQoL on five dimensions with three levels of severity [29]. Utility valuations range between 1.0 (perfect health) to 0.33 (worst state). These EQ-5D-3L scores were used to estimate total QALYs for Landaan and the waitlist control between pre and post-test (approximately seven months) and between post-test and three year follow-up, using the area under the curve method [30]. The method incorporates both the length of time and changes in utilities between the different time points.

### Intervention costs

Costs were collected from a third party payer perspective, which in this study is a local authority. Information from the trial was gathered regarding the (1) necessary training for practitioners, (2) time needed for practitioners to prepare and lead the sessions, and the additional time needed after the sessions, (3) material for participants as well as practitioners and (4) venue required for the group sessions. All resources needed were multiplied by average hourly salaries (for relevant professions) [31], and publicly available rental costs for public venues in the city where the intervention was trailed [32]. Discussions with the researcher who developed *Ladnaan* were held to estimate the potential costs if implemented in the real world. No resource use data were collected alongside the original trial. Costs are reported in 2020 Euro (€).

### Statistical analyses

The time perspective of the following analyses were roughly 7 months, which is the time between baseline and post-test measurements. Total score on the CBCL scale at post-test, as well as QALY changes between pre and post-test, were used as the primary outcomes. Base case analyses were guided by an intention-to-treat principle, including all participants with baseline data (n = 120). For missing data on the outcomes of interest (CBCL and GHQ-12), multiple imputation by chained equations were used [33]. Changes over time between *Ladnaan* and the waitlist control were estimated using generalized linear models, both for count data (CBCL) and continuous outcomes (QALY), using normal and negative binomial distributions and standard link functions respectively. Baseline CBCL and utility scores were controlled for in all analyses [34], and additional covariates were included in the net benefit regression models. Data were cleaned and managed in Excel 2016 and all statistical analyses were performed in R Studio V.3.4.2.

### Net benefit regression to estimate cost-effectiveness

The study employed a net benefit regression framework [35], estimating the expected INB derived from *Ladnaan* in comparison to the waitlist control over a 7 months’ time horizon.

Mathematically, the INB can be defined as

1 $$\begin{aligned} INB\left( {WTP} \right) & = WTP*\left( {\Delta E_{Intervention} - \Delta E_{Control} } \right) - \left( {\Delta C_{Intervention} - \Delta C_{Control} } \right) \\ & = WTP* \Delta E - \Delta C \\ \end{aligned}$$

where ΔE_Intervention_, ΔE_Control_ are changes in effects and ΔC_Intervention_, ΔC_Control_ are changes in costs for the intervention and control group between two time points. Applying the same logic from interpreting ICERs, where an intervention is deemed cost-effective if ICER = ΔC/ΔE < WTP, an intervention would be good value for money if WTP × ΔE > ΔC. Effectively, this is when the INB is positive. However, when there are two outcomes of interest, such as (1) QALYs gained by parents and (2) children’s mental health improvement (CBCL), decision-makers need to define two WTPs, one for each outcome of interest (WTP_1_ and WTP_2_). Hence, Eq. (1) needs to be redefined to

2 $$INB\left( {WTP_{1} ,WTP_{2} } \right) = WTP_{1} *\Delta E_{1} + WTP_{2} *\Delta E_{2} - \Delta C.$$

Concerning the nature of public health interventions, which often build on the various determinants that may affect an individual’s wellbeing, it would be relevant not to view the intervention’s effects in isolation, but rather combined. For example, *Ladnaan*’s goals are to improve child mental health and parents’ quality of life through strengthening parental sense of efficacy and well-being, and inherently, a decision-maker may have two WTPs for the two outcomes (i.e., CBCL and QALY). If we assume that the two outcomes are proportional (e.g., WTP_2_ = *k* * WTP_1_), we can rewrite Eq. (2) as

3 $$\begin{aligned} INB\left( {WTP_{1} ,WTP_{2} } \right) & = \left( {WTP_{1} *\Delta E_{1} } \right) + \left( {k*WTP_{1} *\Delta E_{2} } \right) - \Delta C \\ & = \underbrace {{WTP_{1} \left( {\Delta E_{1} + k*\Delta E_{2} } \right)}}_{{{\text{value}}\;{\text{of}}\;{\text{multiple}}\;{\text{outcomes}}}} - \Delta C. \\ \end{aligned}$$

In Eq. 3, *k* represents the relative weight of WTP_2_ in relation to WTP_1_. This concept is illustrated in Negrín et al. [13]. By using each individual’s (parent or child) net benefit as the dependent variable in a multiple linear regression framework, we can determine if *Ladnaan* is cost-effective at α_1_ > 0, from the following equation

4 $$NB = \alpha_{0} + \alpha_{1} Ladnaan + {\varvec{\alpha}}_{{\varvec{X}}} {\mathbf{X}} + \varepsilon_{nb} ,$$

where ${\varvec{\alpha}}_{{\varvec{X}}}$ is a vector of coefficients for the covariates in the **X** matrix. The estimate of α_1_ equals the INB from Eq. (1) [36]. From Eq. (2), we can test various levels of the two WTPs that render the INB positive, while from Eq. (3), we can assess the relative weight of WTP_2_ with regard to WTP_1_.

Various sensitivity analyses were performed to test how different assumptions made a priori influenced the results. These analyses included: (1) assuming how implementation in a real life setting affect the intervention cost, (2) only including individuals with complete data on the outcomes of interest (n = 79), (3) only including intervention completers, defined as individuals who completed 8 sessions or more (n = 40), (4) only considering baseline values for CBCL and EQ-5D as covariates and (5) using estimates from the follow-up assessment at three years (holding constant the estimates for the waitlist control at post-test) as the main outcome. When analyzing the data from the follow-up assessment, a 3% annual discount rate was applied to the effects, as recommended in Swedish guidelines [37].

## Results

Mean (95% CI) CBCL scores for children of parents participating in *Ladnaan* were 13.67 (10.71–16.64) at baseline and 7.67 (1.28–13.08) at post-test. Respective scores for children of parents in the control group were 10.83 (8.55–13.10) at baseline and 10.01 (4.63–15.40) at post-test. Changes in CBCL scores between baseline and post-test, and baseline and follow-up, were significantly different between the two groups (p < 0.001), favouring the intervention group. For the parent outcome (EQ-5D-3L scores), mean (95% CI) for parents in the intervention was 0.95 (0.94–0.96) at baseline and for the waitlist control 0.96 (0.95–0.96). Unadjusted models showed that at post-test, the additional QALYs generated from the intervention was 0.57 (0.56–0.60), and the corresponding estimate for the waitlist control was 0.57 (0.55–0.60) (over a seven month period). When controlling for baseline utility values, the differences in total QALYs over the trial period were significant at post-test (p < 0.05), favoring the intervention group.

### Intervention costs

The intervention costs ranged between €538 per parent for implementation in a real life setting, spreading staff training costs over a larger number of individuals, and €1589 per parent when divided by intervention completers (attendance > 8 sessions). If spreading costs over all intervention participants in the trial, the cost per parent was €1096. These costs are presented in Table 2.

**Table 2:** Total intervention cost for Ladnaan (Euro 2020)

Item	Quantity	Cost
Training cost		
Training session (time spent by facilitator + trainer)^a^	32	2212
Supervision time needed during the first year (time spent by facilitator + trainer)	10	268
Number of facilitators for the study	9	
Number of facilitators for clinical practice	2	
Total for the study		21,118
Total for implementation in clinical practice^b^		4693
Cost of delivery		
Recruitment session (hours)	1	
Societal information (hours)	6	
Connect sessions (amount)	10	
Time per session for Connect (hours) in the study	1	
Time per session for Connect (hours) outside of the study	2	
Preparation and wrapping-up time per session	8	
Amount of sessions (total)	13	
Facilitators per group	2	
Amount of parents per group	20	
Venue (per hour)		48
Material (per group)		–
Refreshment (per person)		4
Total per group		7069
Total for all groups in the study (n = 58)		42,435
Total for implementation in clinical practice (n = 200)^b^		70,692
Total cost for the study		
Training + delivery		63,553
Cost/parent for the parents that started Ladnaan (n = 58) (training + delivery)*		1096
Cost/parent for those who attended ≥ 8 sessions (n = 40)		1589
Total cost if implemented in normal practice		
Training + delivery		75,385
Cost/parent for those who start		377
Cost/parent for those who will attend ≥ 8 sessions (approx. 70%)^c^		538

The amount of parents that were randomized and started Ladnaan = 58. The amount of parents that would receive the intervention if implemented in clinical practice = 200 (2 facilitators trained per training session, who in total can have 2 groups per year with 20 parents in each, over 5 years’ time)

^a^The cost to attend the training is set at 15,000SEK per person

^b^Estimated based on the max number of children per group (n = 6) and from how many groups can be held by the 20 facilitators yearly (n = 20)

^c^Based on the attendance rate in the trial

^*^Cost used in the base case analysis

### Cost-effectiveness

**Fig. 1:**
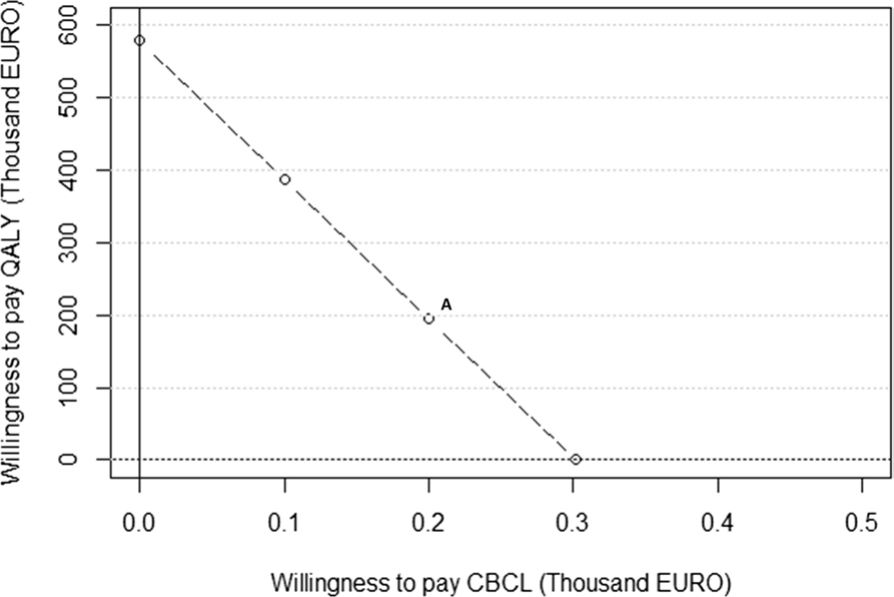
Relationship between willingness-to-pay for a QALY vs. willingness-to-pay for a point improvement on the CBCL

Controlling for baseline outcome values and potential confounders (child and parent age and gender, and parent occupation), *Ladnaan* resulted in 0.002 (95% CI 0.000–0.004) more QALYs per person at post-test (ΔE_1_) in comparison to the waitlist control. At the same time point, Landaan led to a reduction in CBCL total problems score of 2.34 (95% CI 1.22–3.47) points, in relation to the waitlist control (ΔE_2_), controlling for all confounders. These results are depicted in Table 3. With an intervention cost of €1096, a WTP of €302 for one point improvement on the CBCL scale (i.e., WTP_1_ = €302) would yield a positive net benefit (cost-effective result) without considering the value of an additional QALY (i.e., WTP_2_ = 0). Alternatively, WTP_2_ would have to be almost €580,000 to yield a cost-effective result, if an additional point improvement on the CBCL scale was not valued (i.e., WTP_1_ = 0). These results are illustrated in Fig. 1. The dotted line represents values for which INB > 0, which means that for any point above the line, the intervention has at least 50% probability of cost-effectiveness.

**Table 3:** Regression estimates used to estimate the Incremental Net Benefit

Variable	Regression equation	Estimate	95% CI	
Cost, ΔC (in 2020 €)	N/R	1096	–	
Effect, ΔE1 (QALY)	$E_{1A} = \alpha_{0} + \alpha_{1} Ladnaan + \alpha_{2} BaseEQ5D$	0.003	0.000–0.004	
	$E_{1B} = \alpha_{0} + \alpha_{1} Ladnaan + \alpha_{2} BaseEQ5D + \alpha_{3} Confounders$	0.002	0.000–0.004	
Effect, ΔE2 (CBCL)	$E_{2A} = \gamma_{0} + \gamma_{1} Ladnaan + \gamma_{2} BaseCBCL$	− 2.56	− 3.69 to − 1.43	
	$E_{2B} = \gamma_{0} + \gamma_{1} Ladnaan + \gamma_{2} BaseCBCL + \gamma_{3} Confounders$	− 2.34	− 3.47 to − 1.22	

WTP1 (for CBCL)	WTP2 (for QALY)	Regression equation	Estimate	95% CI
0	578,923	$$\begin{gathered} {\text{NB}} = \theta_{0} + \theta_{1} Ladnaan + \theta_{2} Basecost + \theta_{3} BaseCBCL \hfill \\ + \theta_{4} BaseEQ5D + \theta_{5} Confounders + \varepsilon_{nb - p} \hfill \\ \end{gathered}$$	0	− 1153.56 to 1161.40
100	386,816	–	0	− 872.18 to 889.71
200	194,710	–	0	− 680.72 to 650.98
302	0	–	2.34	− 667.78 to 672.47

ΔC: difference in costs between Ladnaan and waitlist control; ΔE1: difference in effects between Ladnaan and waitlist control for QALY at post-test; ΔE2: difference in effects between Ladnaan and waitlist control for CBCL at post-test; ΔNB: difference in net benefit between Ladnaan and waitlist control; Basecost: baseline value of costs; BaseEQ5D: baseline value on the EQ5D scale; BaseCBCL: baseline value on the total CBCL scale; CBCL: Child behaviour Checklist; EQ5D: Euroqol’s five dimension three levels; WTP: Willingness-to-pay

Primary outcome—unit change in total CBCL score for children and QALY gains for parents

Confounders: child and parent age and gender, and parents occupation at baseline

**Fig. 2:**
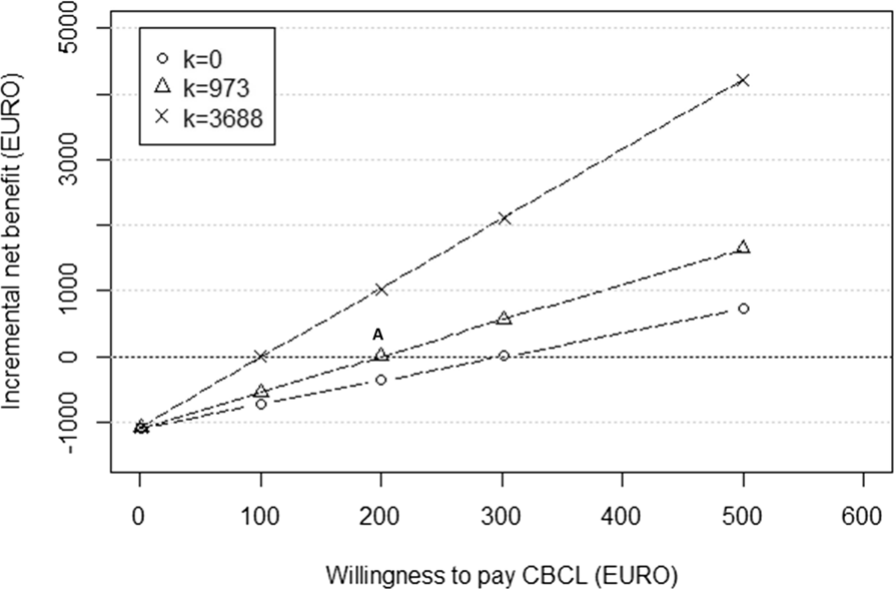
Relationship between the incremental net benefit and willingness-to-pay for a point improvement on the CBCL

Figure 2 presents the results from Fig. 1 in an additional way, using Eq. (3). This graph shows the relationship between WTP for CBCL improvement (a child-based outcome) and WTP for QALYs (a parent-based outcome). More specifically, Fig. 2 illustrates the impact of various assumptions about their relative value (i.e., *k*). For instance, for a positive net benefit and a WTP of €200 for a one-point improvement on the CBCL scale (point A in Fig. 2), we would have to be willing to pay 973 times *more* per QALY, than we are for one point improvement on the CBCL scale. This is the same as point A in Fig. 1, where 973 × €200 = €194,600.

**Fig. 3:**
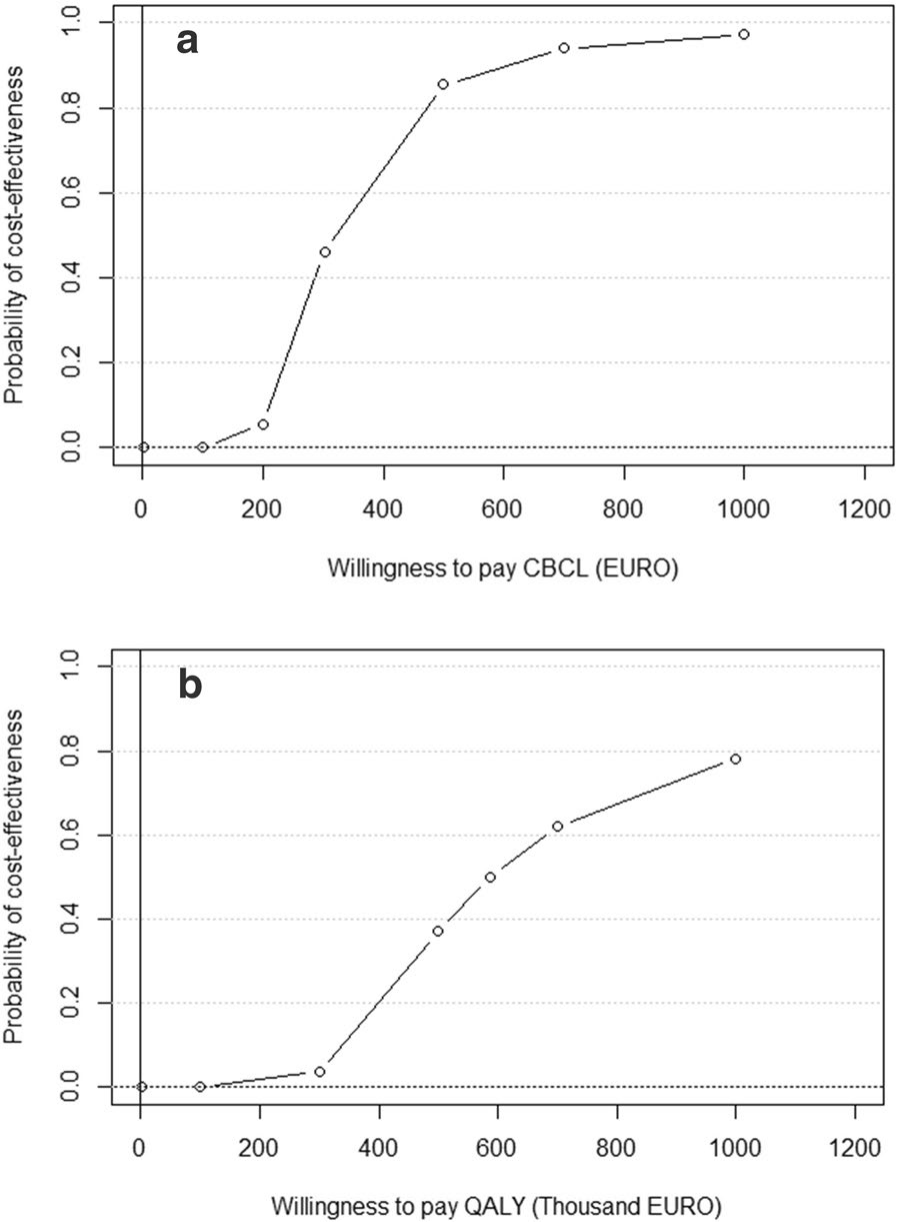
**a** Probability of cost-effectiveness for various amounts of willingness-to-pay for a one-point improvement on the CBCL-scale. **b** Probability of cost-effectiveness for various amounts of willingness-to-pay per QALY

Viewing the cost-effectiveness acceptability curves illustrated in Fig. 3a, b, the lines represent the probability of cost-effectiveness at different WTP. Figure 3a shows that the probability that *Ladnaan* is cost-effective increases to around 97% if the WTP is €1000 for one point reduction in total CBCL problem score. For the parent outcome, presented in Fig. 3b, the probability reaches roughly 78% for a WTP of €1,000,000 per QALY. Although no shadow value for WTP for public health interventions exists in Sweden, there are rates that are recommended for pharmaceuticals. New medication are generally accepted for reimbursement in Sweden at a price of 700,000 SEK (approximately €61,000) per QALY [11]. Given this rate, the intervention would not be cost-effective, when considering only the parental outcome. As there are no shadow values for clinical outcomes, such as the CBCL, it cannot be stated whether the intervention is cost-effective in relation to any *official* WTP.

The intervention was more cost-effective in the follow scenarios: implementation in a real life setting (lower costs); only including intervention completers; and by using the three-year follow-up results (holding constant the estimates for the waitlist control at post-test). However, when we only included intervention completers, the intervention was less cost-effective; the WTPs had to increase for the INB > 0 (WTP_1_ = €1,063,690 and WTP_2_ = €468). When only including baseline values as covariates, the intervention became cost-effective at a lower WTP_2_ (€416,500), but at a slightly higher WTP_1_ (€327).

## Discussion

This paper aimed to assess the cost-effectiveness of a parenting program provided to Somali-born parents, in relation to a waitlist control, by including two different outcomes: one for parents and one for children. The study found that the amount a decision-maker would have to be willing to pay for a one point improvement on the CBCL scale was small, only about €300. However, if looking at HRQoL for parents, the WTP would have to be almost €600,000 per QALY for the intervention to be cost-effective, in relation to a waitlist control. This is because the extra quality of life gain is less than one day (i.e., ΔE < 1/365 = 0.003). When considering multiple outcomes, aggregating results into one graph (with different levels of one WTP in relation to the other) can be informative. Results are robust to key parameter changes. The largest (negative) impact on the results came from only considering individuals who participated in eight or more sessions (maximum amount 12). For these individuals, intervention effects were surprisingly worse, especially for parents. However, the effect differences between intervention completers vs. non-completers were not statistically significant.

Child health promotion interventions are often complex, both in methodology and delivery. Effects may also be widespread, especially when considering caregivers, increasing the difficulty in capturing all when evaluating an intervention. Without properly accounting for these in analyses, or at least acknowledging them in discussions, we run the risk of underestimating the true value of health promotion and disease or problem prevention. The width of effects may stem from the direct effect on multiple sectors if outcomes for one individual improve. It may also be related to indirect effects on other individuals, such as caregivers, and furthermore which sectors they directly influence [1]. In this study, direct effects on different sectors could not be assessed; however, we were able to consider effects on more individuals than primarily targeted. There have been previous attempts to address spillover effects on parents in economic evaluations [9, 38] using different methods to combine QALYs. Often, the interventions had a higher probability of cost-effectiveness when spillovers were included, than if analyses had been done separately. As we were unable to simply add effects, due to the different scales used, we employed another approach to capture spillovers and present them combined with the primary outcome.

In addition, there is scant evidence on the cost-effectiveness of parenting programs on parents with different ethnic backgrounds. Previous research has shown that parenting programs seem to have similar effects on parents, regardless of ethnicity [19]. However, perception of problems may differ [20], and lack of knowledge on how and where to seek help may affect demand for services [24]. Filling the information gap, one of the aims with culturally adapting the parenting program Connect and creating *Ladnaan*, may therefore have contributed to attracting a group that otherwise has low attendance, or simply does not seek help.

A strength of the study was the use of the net benefit regression approach, as it avoids the methodological shortcomings surrounding the ICER estimate that come with standard cost-effectiveness methods [35]. It also allowed for inclusion of two effects (clinical and generic) in the same regression analysis, which is a novel but more importantly practical technique for public health evaluations. However, the study also has limitations. Firstly, no information on resource use was collected during the trial, whereby we have no way to understand if the intervention resulted in an increased or reduced use of public resources. For this specific population who may be less knowledgeable of where and how to seek help, this intervention that aimed to close that knowledge gap may have resulted in an increased use of public services. Another limitation inherited from the trial was the short time-horizon. Although health outcomes were collected three years after intervention delivery, the waitlist control had by then received the intervention and the sustainability of effects are thus difficult to estimate. Another issue relates to the estimation of health utilities, which were derived by mapping GHQ-12 to EQ-5D-3L. It allowed us to capture the intervention effect on different aspects of quality of life, using preference weights derived from a Swedish population [27]. However, information may be lost when mapping, even with good predictive ability. As the EQ-5D-3L has been translated to Swedish and has Swedish social tariffs [28], direct use of it rather than through mapping may have shown different results. A possible drawback is also the use of tariffs from the Swedish population, which may not be representative for Somali-born parents, especially if recently immigrated. Unfortunately, no Somali tariffs are available. The small differences in QALYs found over the trial period may be due to parents rating their general health and thus quality of life as very high at baseline in both the intervention and control group, ranging between 0.95 and 0.96. This is an inherent difficulty when delivering interventions to a normal, healthy population, where we expect low effects (i.e. small improvements). The results ought to be interpreted in light of this consideration. Another limitation with the study stemmed from the lack of Swedish norms for the CBCL, which would have allowed for estimation of a clinical cut-off level. Thus, we were only able to estimate cost per point improvement on the CBCL, which may affect its clinical relevance.

Including multiple effects in the same analysis proves both possible and may be highly relevant for decision-makers considering widespread effects of interventions, especially since national guidelines in several countries recommend that evaluations take a societal perspective, and include spillover effects [39, 40]. Both parents and children benefited from the intervention, but effects differed, which may lead to different investment decisions if looked at in isolation. Implementation of *Ladnaan* has in previous studies proven to be effective with regards to multiple outcomes for children [23] and parents [22]. Whether the effects are worth investment in is ultimately a decision for, in this case, local authorities, in relation to their priorities. The lowest amount needed for the intervention to generate a positive net benefit, in relation to a waitlist control, is roughly €300. This may be put in relation to the potentially much larger financial impact on society if child problems persist, especially if concurrence with parental ill-health [6, 7], or if they continue into adulthood.

## Conclusion

A cultural adaptation of a parenting program is beneficial for parents and children. Further, allowing for both child and parent effects to be analysed simultaneously in relation to intervention costs is relevant when incorporating spillover effects in decision-making. The amount a decision-maker would have to be willing to invest in a parenting intervention for Somali born parents depends on which effects she is willing to consider and their relative priority in relation to budget considerations.

## Data Availability

The datasets used and/or analysed during the current study are available from the corresponding author on reasonable request.

## Abbreviations

CBCL: Child Behavior Checklist
EQ-5D-3L: Euroqol’s five dimension three level scale
GHQ-12: General Health Questionnaire
HRQoL: Health related quality of life
ICER: Incremental cost-effectiveness ratio
INB: Incremental net benefit
QALY: Quality adjusted life years
WTP: Willingness to pay

## References

[1] Brouwer WBF, Van Exel NJA, Tilford JM (2010). Incorporating caregiver and family effects in economic evaluations of child health. Economic evaluation in child health.

[2] Wittenberg E, Ritter GA, Prosser LA (2013). Evidence of spillover of illness among household members: EQ-5D scores from a US sample. Med Decis Mak.

[3] Weissman MM, Wickramaratne P, Nomura Y, Warner V, Pilowsky D, Verdeli H (2006). Offspring of depressed parents: 20 years later. Am J Psychiatry.

[4] Grimbos T, Granic I (2009). Changes in maternal depression are associated with MST outcomes for adolescents with co-occurring externalizing and internalizing problems. J Adolesc.

[5] Marchand JF, Hock E, Widaman K (2002). Mutual relations between mothers’ depressive symptoms and hostile-controlling behavior and young children’s externalizing and internalizing behavior problems. Parenting.

[6] Knapp M, Wong G (2020). Economics and mental health: the current scenario. World Psychiatry.

[7] Nystrand C, Ssegonja R, Sampaio F (2019). Quality of life and service use amongst parents of young children: results from the children and parents in focus trial. Scand J Public Health.

[8] Prosser LA, Wittenberg E (2019). Advances in methods and novel applications for measuring family spillover effects of illness. Pharmacoeconomics.

[9] Tubeuf S, Saloniki E-C, Cottrell D (2019). Parental health spillover in cost-effectiveness analysis: evidence from self-harming adolescents in England. Pharmacoeconomics.

[10] National Institute for Health and Care Excellence (NICE) (2012). Methods for the development of NICE public health guidance.

[11] Svensson M, Nilsson FOL, Arnberg K (2015). Reimbursement decisions for pharmaceuticals in Sweden: the impact of disease severity and cost effectiveness. Pharmacoeconomics.

[12] WHO (2003). WHO making choices in health: WHO guide to cost-effectiveness analysis.

[13] Negrín MA, Vázquez-Polo FJ (2006). Bayesian cost-effectiveness analysis with two measures of effectiveness: the cost-effectiveness acceptability plane. Health Econ.

[14] Salari R, Enebrink P, Sanders MR, Morawska A (2018). Role of universal parenting programs in prevention. Handbook of parenting and child development across the lifespan.

[15] Barlow J, Coren E (2018). The effectiveness of parenting programs: a review of campbell reviews. Res Soc Work Pract.

[16] Alfredsson EK, Thorvaldsson V, Axberg U, Broberg AG (2018). Parenting programs during adolescence: outcomes from universal and targeted interventions offered in real-world settings. Scand J Psychol.

[17] Chu JTW, Bullen P, Farruggia SP, Dittman CK, Sanders MR (2015). Parent and adolescent effects of a universal group program for the parenting of adolescents. Prev Sci.

[18] Moretti MM, Obsuth I (2009). Effectiveness of an attachment-focused manualized intervention for parents of teens at risk for aggressive behaviour: the connect program. J Adolesc.

[19] Leijten P, Raaijmakers MAJ, Orobio de Castro B, Matthys W (2016). Ethnic differences in problem perception: immigrant mothers in a parenting intervention to reduce disruptive child behavior. Am J Orthopsychiatry.

[20] Leijten P, Raaijmakers MAJ, Orobio de Castro B, van den Ban E, Matthys W (2017). Effectiveness of the incredible years parenting program for families with socioeconomically disadvantaged and ethnic minority backgrounds. J Clin Child Adolesc Psychol.

[21] Moretti MM, Braber K, Obsuth I (2013). Connect: an attachment-focused treatment group for parents and caregivers—a principle based manual.

[22] Osman F, Salari R, Klingberg-Allvin M, Schön U-K, Flacking R (2017). Effects of a culturally tailored parenting support programme in Somali-born parents’ mental health and sense of competence in parenting: a randomised controlled trial. BMJ Open.

[23] Osman F, Flacking R, Schön U-K, Klingberg-Allvin M (2017). A support program for Somali-born parents on children’s behavioral problems. Pediatrics.

[24] Osman F, Klingberg-Allvin M, Flacking R, Schön U-K (2016). Parenthood in transition—Somali-born parents’ experiences of and needs for parenting support programmes. BMC Int Health Hum Rights.

[25] Achenbach TM, Dumenci L (2001). Advances in empirically based assessment: revised cross-informant syndromes and new DSM-oriented scales for the CBCL, YSR, and TRF: comment on Lengua, Sadowksi, Friedrich, and Fischer. J Consult Clin Psychol.

[26] Goldberg D, Williamsn P (1988). A user’s guide to the General Health Questionnaire.

[27] Lindkvist M, Feldman I (2016). Assessing outcomes for cost-utility analysis in mental health interventions: mapping mental health specific outcome measure GHQ-12 onto EQ-5D-3L. Health Qual Life Outcomes.

[28] Burstrom K, Sun S, Gerdtham UG, Henriksson M, Johannesson M, Levin LA, Zethraeus N (2014). Swedish experience-based value sets for EQ-5D health states. Qual Life Res.

[29] Rabin R, de Charro F (2001). EQ-5D: a measure of health status from the EuroQol Group. Ann Med.

[30] Matthews JN, Altman DG, Campbell MJ, Royston P (1990). Analysis of serial measurements in medical research. BMJ.

[31] Statistics Sweden (Statistiska Centralbyrån). Statistics on salaries for munisipality employees (Konjunkturstatistik, löner för kommuner). 2020. https://www.scb.se/hitta-statistik/statistik-efter-amne/arbetsmarknad/loner-och-arbetskostnader/konjunkturstatistik-loner-for-kommuner-klk/. Accessed 24 Feb 2020.

[32] Borlänge Municipality. Rental costs for public venues in Borlänge municipality. 2020. https://www.borlange.se/uppleva-och-gora/foreningar-foreningsliv/boka-lokal-och-anlaggning. Accessed 24 Feb 2020.

[33] Buuren S, Groothuis-Oudshoorn C (2011). MICE: multivariate imputation by chained equations in R. J Stat Softw.

[34] Manca A, Hawkins N, Sculpher MJ (2005). Estimating mean QALYs in trial-based cost-effectiveness analysis: the importance of controlling for baseline utility. Health Econ.

[35] Hoch JS, Briggs AH, Willan AR (2002). Something old, something new, something borrowed, something blue: a framework for the marriage of health econometrics and cost-effectiveness analysis. Health Econ.

[36] Hoch JS, Dewa CS (2007). Lessons from trial-based cost-effectiveness analyses of mental health interventions: why uncertainty about the outcome, estimate and willingness to pay matters. Pharmacoeconomics.

[37] The Dental and Pharmaceutical Benefits Agency (TLV) (2017). General guidelines for economic evaluations.

[38] Ulfsdotter M, Lindberg L, Månsdotter A (2015). A cost-effectiveness analysis of the Swedish universal parenting program all children in focus. PLoS ONE.

[39] National Healthcare Institute. Guideline for economic evaluations in healthcare. 2016.

[40] Sanders GD, Neumann PJ, Basu A (2016). Recommendations for conduct, methodological practices, and reporting of cost-effectiveness analyses: second panel on cost-effectiveness in health and medicine. JAMA.

